# Bakuchiol and its pharmacological benefits

**DOI:** 10.12688/f1000research.129072.1

**Published:** 2023-01-09

**Authors:** Nuder Nower Nizam, Sohel Mahmud, Mohammad Kamruzzaman, Md. Kamrul Hasan

**Affiliations:** 1 Department of Public Health, American International University Bangladesh, Dhaka, 1229, Bangladesh; 2 Department of Biochemistry and Molecular Biology, Tajgaon College, Dhaka, National University, Bangladesh, Gazipur, 1704, Bangladesh; 3 Department of Biochemistry and Molecular Biology, University of Dhaka, Dhaka, 1000, Bangladesh; 4 Department of Public Health, North South University, Dhaka, 1229, Bangladesh

**Keywords:** Bakuchiol, Psoralea corylifolia, medicinal plants, health benefits

## Abstract

**Background and aims:** Natural compounds extracted from medicinal plants have recently gained attention in therapeutics as they are considered to have lower toxicity and higher tolerability relative to chemically synthesized compounds. Bakuchiol is one such compound; it is a type of meroterpene derived from the leaves and seeds of *Psoralea corylifolia* plants. Natural sources of bakuchiol have been used in traditional Chinese and Indian medicine for centuries due to its preventive benefits against tumors and inflammation. It plays a strong potential role as an antioxidant with impressive abilities to remove Reactive Oxygen Species (ROS). This review has focused on bakuchiol’s extraction, therapeutic applications, and pharmacological benefits.

**Methods:** A search strategy has been followed to retrieve the relevant newly published literature on the pharmacological benefits of bakuchiol. After an extensive study of the retrieved articles and maintaining the inclusion and exclusion criteria, 106 articles were finally selected for this review.

**Results:** Strong support of primary research on the protective effects via antitumorigenic, anti-inflammatory, antioxidative, antimicrobial, and antiviral activities are delineated.

**Conclusions:** From ancient to modern life, medicinal plants have always been drawing the attention of human beings to alleviate ailments for a healthy and balanced lifestyle. This review is a comprehensive approach to highlighting bona fide essential pharmacological benefits and mechanism of action of therapeutic implications.

**List of abbreviations**
3-NT: 3 Nitrotyrosine
4HNE: 4-Hydroxynonenal
AKT: Protein kinase B
AMPK: AMP-activated protein kinase
AR: Androgen receptor
ATM: Ataxia-Telangiectasia Mutated Kinase
ATM: ATM serine/threonine kinase
BAK: Bakuchiol
Bax: Bcl-2-associated X protein
Bcl2: B-cell lymphoma 2
Cdc2: Cell division control 2
Col I: Collagen type I
Col III: Collagen type III
COX2: Cyclooxygenase2
ER-alpha: Estrogen Receptor alpha
ER-beta: Estrogen Receptor beta
GSH-Pxi: Inducible Glutathione peroxidase
GSTA3: Glutathione S-Transferase Alpha 3
HIF-1: Hypoxia-inducible factor 1
H1N1: Hemagglutinin 1 neuraminidase 1
HNO _3_
: Nitric acid
IL6: Interleukin 6
iNOS: Inducible Nitric Oxide Synthase
LTB4: Leukotriene B4
LPS: Lipopolysaccharide
MDA: Malondialdehyde
MAPK: Mitogen-activated protein kinases
MMP-1: Matrix metalloproteinase-1
Myt1: Myelin Transcription Factor 1
NFkB: Nuclear Factor kappa-light-chain-enhancer of activated B cells
NQ01: NAD(P)H Quinone Dehydrogenase 1
NSCLC: Non-small cell lung cancer
Nrf1: Nuclear Respiratory Factor 1
p38: Tumor protein p53
PGC-1α: Proliferator-activated receptor gamma coactivator 1-alpha
PGE2: Prostaglandin E2
P-Cdc2: Phosphorylated cell division cycle protein 2
Rac: Ras-related C3 botulinum toxin
ROS: Reactive Oxygen Species
SARS-CoV-2: Severe Acute Respiratory Syndrome-CoronaVirus-2
Sirt1: NAD-dependent deacetylase sirtuin-1
STAT3: Signal transducer and activator of transcription 3
SOD: Superoxide Dismutase
TIMP-1: Tissue inhibitors of metalloproteinases-1
TIMP-2: Tissue inhibitors of metalloproteinases-2
TNFα: Tumour Necrosis Factor alpha
Trx1: Thioredoxin 1
TXNIP: Thioredoxin-interacting protein

## Introduction

Plants have been used in traditional Indian and Oriental medicine for centuries. Despite improved access to essential medicines as supported by World Health Organization (WHO), ^1^ a large population, especially in less developed countries, still relies on plant-based medications for primary health care needs due to ease of availability and high benefit to cost ratio. ^2^ There is an increase in global demand for medicinal plants due to improved quality of life. Some compounds have been extracted from these beneficial plants to study their mechanisms of action that facilitate their beneficial effects.

Bakuchiol, extracted predominantly and traditionally from the *Psoralea corylifolia L.* plant, is one such compound. It is a meroterpene that has shown potent antimicrobial, anti-inflammatory, anti-osteoporotic, and antitumorigenic activities and exhibited other beneficial uses. ^3^ It has also gained huge popularity in the beauty industry as a tolerable analogue of retinol in skin therapeutics. ^4^ Studies have also shown bakuchiol’s protective effect on the liver, heart, bones, and other organs. ^5^ Recent studies have focused on bakuchiol and its therapeutic effects, showing promise as a multi-targeting therapeutic agent. ^6^^–^^13^

**Figure 1.**
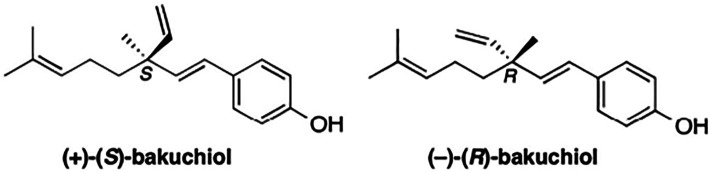
Chemical Structures of naturally occurring (+)-(S)-Bakuchiol and its enantiomer (R)-(-)-Bakuchiol. This figure has been adapted from Khuranna *et al*. ^14^ under CC BY 4.0.

Bakuchiol is a naturally occurring prenylated phenolic isoprenoid ( Figure 1). It is a type of meroterpene with a chiral tetra-alkylated (all-carbon) quaternary center. ^15^ It was first extracted from the seeds of *Psoralea corylifolia* in 1966 when its plane structure was also determined, while its configuration of the quaternary center was determined in 1973. ^16^ The formal chemical name of bakuchiol is 4-[(1E,3S)-3-ethenyl-3,7-dimethyl-1,6-octadien-1-yl]-phenol. Its naturally occurring form (S)-(+)-Bakuchiol has an enantiomer (R)-(-)-Bakuchiol, and its chirality influences their actions and effectiveness.

The source of bakuchiol varies between regions; it is commonly extracted from the seeds and leaves of *Psoralea corylifolia* (Babchi plant) in India, which belongs to the Leguminosae plant family, and is a major phytochemical present in the root and stem of *Ulmus davidiana var.* ^17^ *Japonica* (Japanese Elm) is widely distributed in China, Japan, and Korea. ^6^ These plants have been used in their regions of wide distribution in traditional medicine to treat inflammatory disorders and cancer. *Piper longum* (Long pepper) *, Psoralea glandulosa L, Otholobium pubescens*, *Prosopis glandulosa, Aerva sanguinolenta, Psoralidium tenuiflorum, Pimelea drupacea, Bridelia retusa, Spiraea formosana, Elaeagnus bockii* and other sources of bakuchiol have been discovered in recent times. ^18^^–^^20^

Medicinal plants e.g.: *Psoralea corylifolia* where bakuchiol is derived have been used directly as traditional medication or via pharmaceutical preparations in modern medicine. Recent times have shown collective evidence highlighting the immense potential of traditionally used medicinal plants and their derived compounds. ^21^ An increase in international demand and trade has also been observed, appreciating its low costs and tolerability. In addition, an increasing trend of antimicrobial resistance has been observed due to inappropriate dosage and lack of regulation of prescriptions. It is thus imperative to find new sources of antiviral and antimicrobial therapy to account for the increased resistance observed. Plants and phytochemicals are generally considered sources of tolerable, less toxic treatments. ^21^ Bakuchiol has recently been studied in detail in research settings while being used in traditional medicine for centuries. ^22^

**Figure 2.**
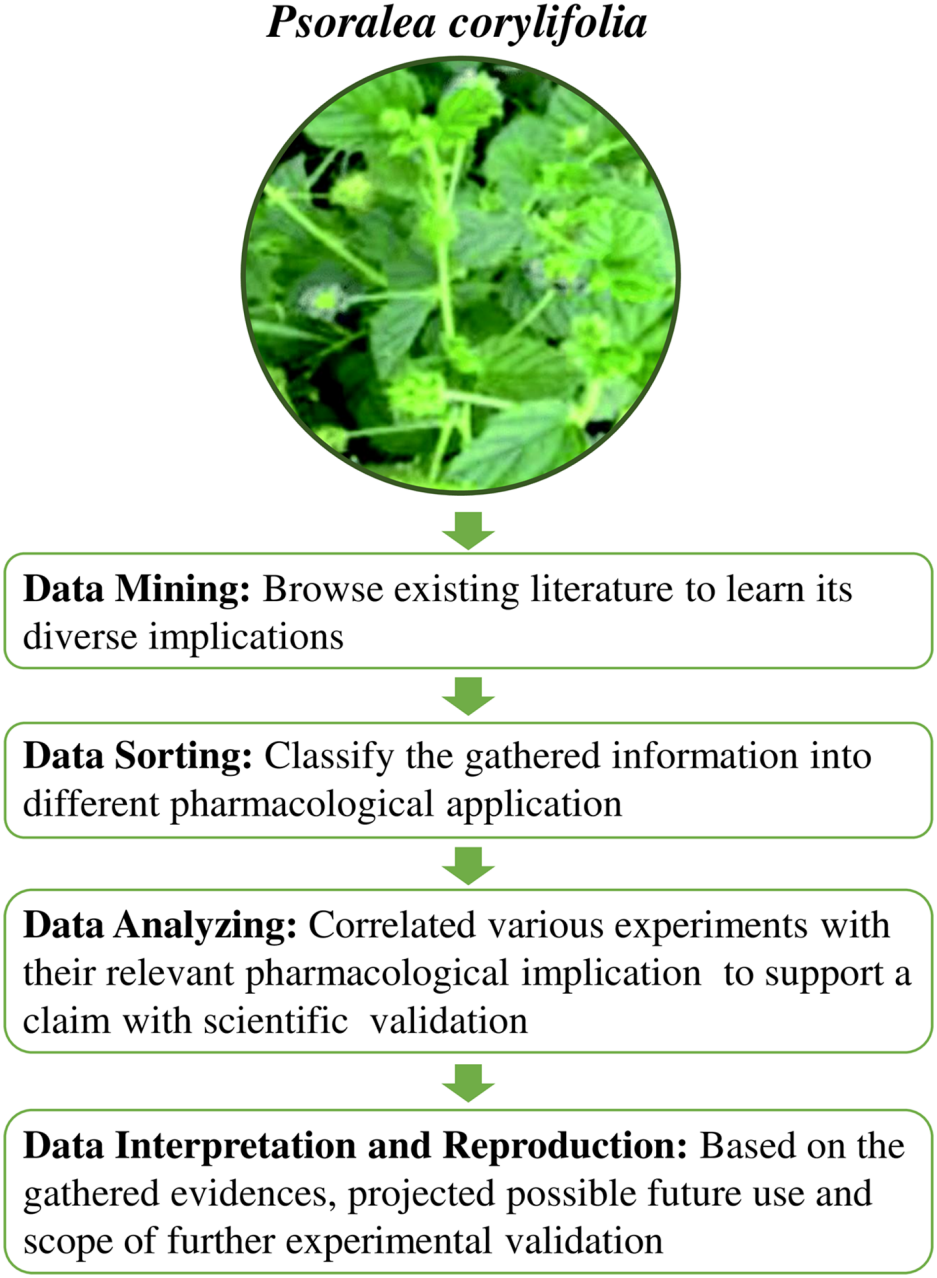
Workflow of the study. This figure is an original figure produced by the author(s) for this review article. The image of *Psoralea corylifolia* has been reproduced from Niu *et al*. ^23^ under CC BY-NC 3.0.

This review article focuses on the potential pharmacological benefits of bakuchiol, focusing on its promising protective effects in controlling activities that lead to the amelioration of non-communicable diseases, as well as verifying its function as an antimicrobial and antiviral agent. The workflow of this review is illustrated in Figure 2. The knowledge gained from this review will shed light on the published research to pave the path for future research perspectives and considerations.

## Methods

### Retrieval of the published articles

This review evaluates the pharmacological uses of bakuchiol, focusing on its promising protective effects on controlling both communicable and non-communicable diseases. Data for this study have been amalgamated from both primary and secondary data resources, including clinical trials (both randomized and non-randomized), as well as *in vitro* and *in vivo* studies, which have evaluated the use of bakuchiol as a potential pharmacological agent in controlling multiple disease conditions.

Several inclusion and exclusion criteria were fixed while selecting articles for this review. The works done from 1990 to 2022 have been studied rigorously. Literature searches were done using the keywords such as “ *Psoralea corylifolia* AND/OR Babchi AND/OR Bakuchiol” in various available online scientific databases. Articles that represent phytochemistry AND/OR pharmacological activity AND/OR health benefits of Bakuchiol published in PubMed, Web of Science, PMC, Google Scholar, ScienceDirect, and ResearchGate were incorporated in this review. From the evaluation of 106 articles, the effect of bakuchiol as a potential antitumorigenic, anti-inflammatory, antioxidative, antimicrobial, and antiviral agents, as characterized in several studies, was finally assessed for this review. In addition, clinical trial databases were also searched to find current registered clinical trials of the use of bakuchiol in multiple disorders, including diabetes, inflammatory disorders of the skin and other organs, oral disorders, cancer, and coronavirus disease 2019 (COVID-19). Only articles published in English were considered for this review. The databases were searched in a timeline from 2010 to date, except for articles related to its initial discovery and extraction, and only approved and published data were considered for this review unless otherwise mentioned. In most studies, the outcomes measured and considered for this review include the severity and comparative assessment of disease progression in bakuchiol-treated and untreated groups.

## Results

### Extraction and solubility of Bakuchiol

Traditional plant-based medications work by grinding the interest portion and then extracting it into carrier oils or spirits. ^24^ However, specific compounds cannot be distilled this way and there may also be the presence of potentially cytotoxic compounds in an extract made in traditional methods. ^25^^,^^26^ While beneficial, the number of bioactive ingredients present in natural sources is generally meager, and extraction processes are time-consuming and lab-intensive, hindering the mass-scale use of natural products in drug selection and development. Current extraction methods include distillation, cold pressing for oils, and solvent extraction, the most commonly used method. ^27^

Factors that affect the ease of solubility and diffusivity are considered during extraction processes, including consideration of the properties of the solvent being used, such as the laws of inter miscibility as well as cost and safety, the size of the raw materials material being used, temperature and time duration. ^27^ Generally, methanol and ethanol are used in solvent extraction for phytochemicals. Smaller particle sizes of raw materials are preferred for better extraction efficiency. ^28^ Temperature is also an essential factor in controlling the extraction rate, as higher temperatures increase solubility and diffusion, but this is not ideal for highly volatile compounds as higher temperatures can cause the decomposition of thermolabile compounds. ^29^

Bakuchiol is soluble in organic solvents such as ethanol and dimethyl sulfoxide (DMSO) and sparingly soluble in aqueous solutions. ^6^ Its solubility can be increased by first making a stock solution with ethanol and then diluting it with aqueous buffers for experimental purposes. The most commonly reported method is extraction with 80% ethanol, followed by silica column chromatography. ^6^ This method is also suitable for deriving other phenolic phytochemicals present in the sources of bakuchiol.

The first synthesis of the naturally occurring form of bakuchiol was done by Carnduff and Miller in 1967, with a Claisen rearrangement being a crucial part of the synthesis process; however, this was not enantioselective. ^20^ Other variations in chemical synthesis methods have also been developed over the years, but current methods focus on concisely synthesizing enantioselective (S)-(+)-Bakuchiol. It is also synthesized chemically in four steps from (E)-geranic acid under aldol reaction conditions, with an overall yield of 53%, increasing commercial availability. ^30^ Stock solutions of (S)-(+)-Bakuchiol synthesized from (E)-geranic acid can be made by dissolving the compound in DMSO.

However, conventional extraction methods require a large solvent volume and longer durations. Separation techniques have developed and come a long way. More sophisticated techniques, including various forms of chromatography, have been developed to extract specific compounds from the plant source. ^14^ Other methods (supercritical fluid extraction, pressurized liquid extraction, and microwave-assisted extraction) with much shorter extraction time and lower solvent consumption may be considered for mass extraction of bakuchiol. ^14^ They may be used on a large scale for cheaper drug development. These methods have also been used in the extractions of natural products, so their efficiency in the extraction of bakuchiol needs to be evaluated. ^31^

Methods are being studied to increase the bioavailability and absorption of bakuchiol and its sources. Cho *et al*. (2011) studied the stability and physicochemical property of *P. corylifolia* extract encapsulated in 3 different vesicles (liposome, niosome, and transfersome) in nude mouse skin. ^32^ The results of this study suggest that the use of niosome and transfersome could be a good bioavailability enhancement system (BAES) for *P. corylifolia* extract to improve skin permeation and stability, highlighting the importance of finding methods to increase the bioavailability of bakuchiol and other phenolic phytochemicals. ^32^

### Therapeutic applications of bakuchiol

**Figure 3.**
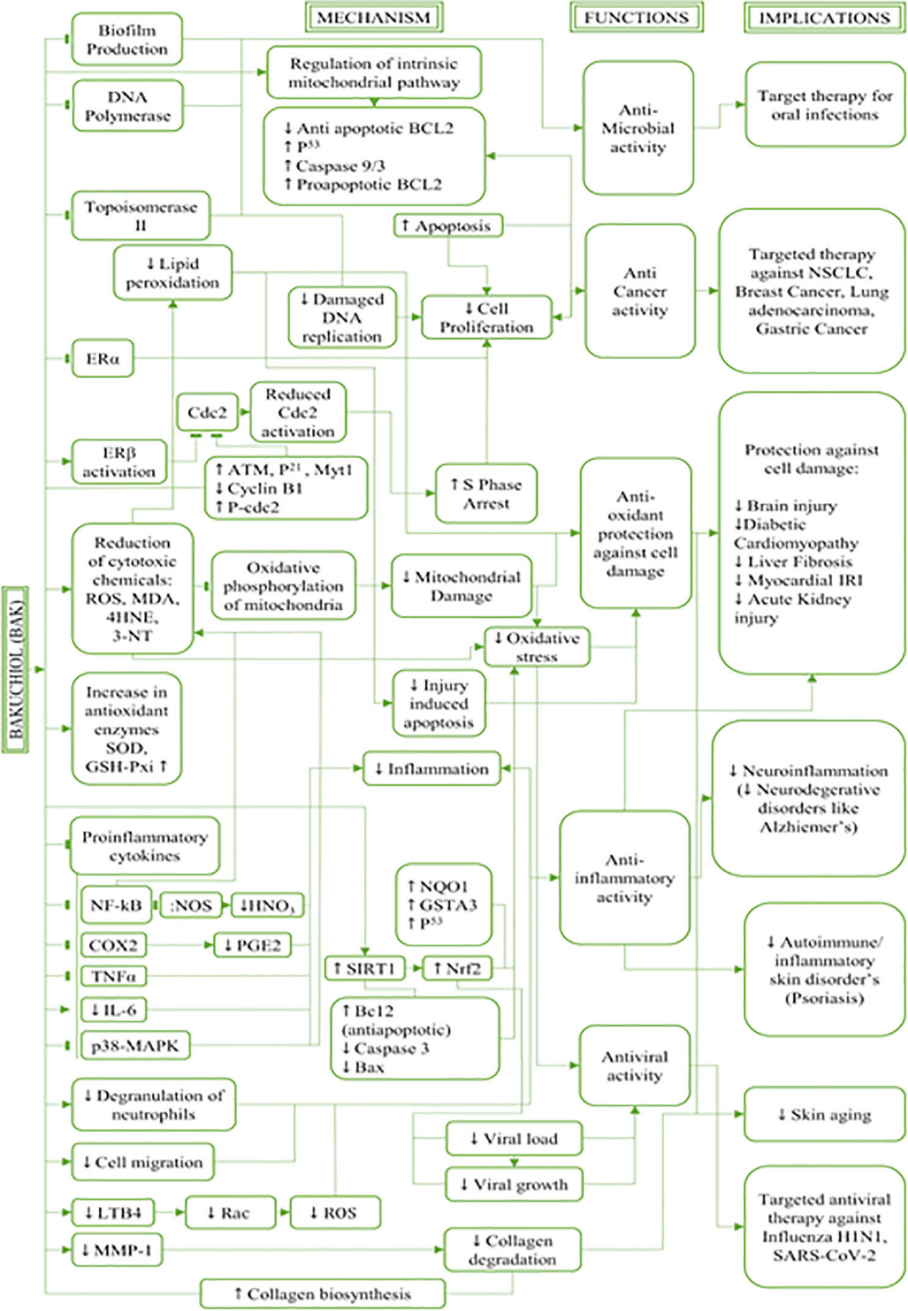
Mechanism of actions, functions, and uses of the naturally occurring compound bakuchiol and its implications in therapeutic approaches. This figure is an original figure produced by the author(s) for this review article.

A large number of studies have evaluated the role of various phytochemicals and their pharmacological applications in communicable and non-communicable diseases. ^33^ In addition, other potentially beneficial compounds have also been extracted from the plant sources of bakuchiol. ^31^ However, since this review focuses on bakuchiol and its pharmacological benefits, other phytochemicals and compounds will not be reviewed in detail and will only be referred to as appropriate. A brief description of the extraction and solubility of bakuchiol precedes the comprehensive analyses of its various pharmacological benefits. The mechanism of actions, functions, and uses of the naturally occurring compound bakuchiol and its implications in therapeutic approaches are described below and illustrated in Figure 3.

***Anti-Inflammatory effects of bakuchiol***

Inflammation is generally caused by the production of pro-inflammatory mediators and cytokines such as nitric acid, prostaglandin E2 (PGE2), tumor necrosis factor-alpha (TNF-α), and interleukin 6 (IL-6). ^34^ The anti-inflammatory effects of bakuchiol have been evaluated in multiple studies, and it has been found that the anti-inflammatory effects of bakuchiol work via various mechanisms. ^7^ It can inhibit the degranulation of neutrophils and decrease cell migration and myeloperoxidase activity (involved in producing oxidants such as hypochlorous acid) in inflammatory sites, generating multiple mechanisms of controlling leukocyte function and inflammation in various types of cells. ^35^

Studies have reported the ability of bakuchiol to inhibit nitric acid and PGE2 production, both pro-inflammatory mediators generated by inducible enzymes nitric oxide synthase and cyclooxygenase-2. ^6^^–^^8^ A concentration of 50μM of bakuchiol has shown a reduction in nitric acid and PGE2 in macrophages by over 50% without exhibiting cytotoxicity. ^6^ Bakuchiol can inhibit the expression of nitric acid synthase gene via the inactivation of nuclear factor kappa-light-chain-enhancer of activated B cells (NFkB), which can inhibit nitric acid production and IL-6 induced signal transducer and activator of transcription 3 **(**STAT3) activation, inhibiting IL-6 production in multiple cell lines. ^36^ This is comparable to other natural compounds, such as aurantiamide acetate from the roots of the *Baphicacanthus cusia.* ^37^

Bakuchiol can suppress inflammatory responses via downregulation of the p38 MAPK/ERK signaling pathway in microglia cells controlling neuroinflammation. ^7^ Moreover, it can reduce the production of leukotriene B4 (LTB4), which contributes to disease severity in chronic inflammatory diseases in a dose-dependent manner. ^38^ LTB4 can generate reactive oxygen species (ROS) via a Rac-dependent pathway, and a reduction in LTB4 production also reduces ROS production. ^39^ Bakuchiol and its derivatives have also exhibited inhibitory activities in lipopolysaccharide (LPS) induced NO production in macrophages. ^40^

Bakuchiol decreases the phosphorylation of ERK, p38, and NF-κB. It arrests the mRNA expression of pro-inflammatory molecules, including TNF-α and interleukin-1β (IL-1β), and inducible nitric oxide synthases (iNOS) in Methylglyoxal-administered mice, which reduces inflammation and helps in the prevention and control of diabetes. ^41^

Altogether, bakuchiol can provide beneficial therapeutic implications in treating inflammatory diseases such as nephritis, asthma, diabetes, skin inflammatory conditions such as psoriasis, and neurodegenerative disorders such as Alzheimer’s. ^40^^–^^42^

***Antimicrobial effects of bakuchiol***

Nature provides a great potential source for antimicrobial drug discovery. Phytochemicals extracted from natural sources are considered to have fewer side effects and a variety of functions, and some exhibit antimicrobial activity. In addition, natural compounds are much more accessible than synthetic compounds available for treatment; most developing countries depend on plant-based medications as the first line of treatment. ^43^

Terpenes such as thymol, carvacrol, eugenol, and menthol, most commonly used in fragrances and aromatherapy, show broad-spectrum antimicrobial activity through efflux pump inhibition and inhibition of bacterial growth. ^44^^–^^46^ Bakuchiol is also a terpene that has recently been characterized to have multi-beneficiary effects on disease control, both communicable and non-communicable.

The antimicrobial effects of bakuchiol have been studied as a natural source of oral healthcare, and multiple studies have shown potent antimicrobial activity. ^11^^,^^47^^,^^48^ Studies evaluating bakuchiol’s antimicrobial effect have shown potent antibiotic effects against *Streptococcus aureus.* Another study examining the antimicrobial effects of bakuchiol against *Streptococcus mutans* showed increased inhibition in the growth of colonies in a dose-dependent manner. Similar results were observed for *Streptococcus sanguis, Streptococcus salivarius, Streptococcus sobrinus, Enterococcus faecalis, Enterococcus faecium, Lactobacillus acidophilus, Lactobacillus casei, Lactobacillus casei, Lactobacillus plantarum, Actinomyces viscosus, Porphyromonas gingivalis* and other bacterial strains that contribute to oral diseases. ^11^

Conventional treatments for oral infections usually contain chlorhexidine, a gold standard. ^49^ However, as it was toxic in large quantities and caused discoloration of the tooth surface, other natural-based alternatives were evaluated, some of which were successful inhibitors for antimicrobial growth. ^50^^–^^53^ There is also a concern about emerging antimicrobial resistance, which has increased rapidly in the past decade and is now one of the leading causes of death. ^54^^,^^55^ Given the increasing emergence of antimicrobial drug-resistant strains, finding nature-based alternatives to antibiotics is vital to help control the resistance.

Bakuchiol works by rupturing the cell membrane in bacterial strains, inhibiting DNA polymerase and other DNA replication enzymes, and reducing biofilm production by *E. faecalis* and *E. faecium.* ^56^ These studies implicate the potential uses of bakuchiol in maintaining oral health care. It can potentially be used in combination with other natural pre-existing methods of oral healthcare to target the increasing demand in the market for natural healthcare products.

Bakuchiol also has antifungal effects, and ethanol isolates of bakuchiol from PF effectively inhibit the growth of dermatophytes *in vitro.* ^57^ Oral *Candida* species have also been susceptible to bakuchiol treatment with minimum inhibitory concentration (MIC) ranging from 12.5 to 100 μg/mL, causing a reduction in growth rates, viable counts, cell metabolic activity and biofilm mass. ^58^

***Bakuchiol in the treatment of skin disorders***

Bakuchiol has recently gained mass popularity as an active component in skin care products due to its functional similarity with retinoid compounds and relatively higher tolerability. ^59^ It has been shown to work with melatonin and vitamin C derivatives to regulate genes involved in the modulation of angiogenesis, collagen biosynthesis, skin barrier function, and other aspects of skin cell biology. ^9^ Bakuchiol also has retinol-like properties in modulating genes that regulate extracellular matrix production and the dermal-epidermal junction. ^60^ It can help deter skin aging by enhancing human fibroblast cell activity and inhibiting the expression of matrix metalloproteinases by increasing tissue inhibitors of metalloproteinases-1 **(**TIMP-1), tissue inhibitors of metalloproteinases-2 **(**TIMP-2), collagen type I (Col I), and collagen type III (Col III) mRNA, and decreasing the expression of matrix metalloproteinase-1 (MMP-1) mRNA. ^61^

Bakuchiol exhibits retinol-like functionality causing upregulation of types I, III, and IV collagen, which make up the skin’s extracellular matrix and basement membrane, in *in vitro* models. ^4^ When formulated into a skincare product, regularly applied, and tested under clinical settings for 12 weeks, significant improvement in facial fine lines and wrinkles were observed, along with improvements in skin elasticity, firmness, and pigmentation, as well as a reduction in photodamage. ^4^ This effect was observed in multiple studies on participants of varying races. ^4^^,^^59^ In addition, other studies have also confirmed an increase in moisture retention from the regular application of products formulated with bakuchiol extract. ^62^ It also works synergistically with other natural compounds to reduce inflammation and protect and maintain naturally aging skin. ^10^

This higher tolerability allows bakuchiol to be an effective anti-aging treatment for people of all skin types. Furthermore, it can also be formulated for people with hyperpigmentation historically perceived as less tolerant of retinol. ^63^

Unlike retinoids, bakuchiol can be used daily due to its photostability. ^59^ Combinations of retinoid and bakuchiol therapy also reduce oxidative stress induced by retinoids, ^64^ as bakuchiol helps to stabilize retinol under photo-oxidative environments.

Bakuchiol works as an antioxidative agent due to its ability to interfere with free radical production, decrease the translocation of mitochondrial apoptosis-inducing factors, and its ability to quench superoxides and other radicals *in vitro.* ^13^^,^^49^ Overall, it can be deduced that its naturally occurring phytochemical functions as a potent antioxidant that can help maintain cellular turnover and ensure protective functions for all organs. With higher tolerability, photostability, and ability to slow down skin aging, bakuchiol can be considered a suitable plant-based alternative to retinol, traditionally extracted from animal sources.

***Antitumorigenic effects of Bakuchiol and Hormone replacement therapy***

Bakuchiol has traditionally been used in medicine for the treatment of various cancers. Early studies have shown that bakuchiol can inhibit cell proliferation, and further studies have recently evaluated its mechanisms of action in various cancer cell lines compared to its analogue resveratrol. ^12^ It can induce ROS-related apoptosis in lung adenocarcinoma A549 cell line, S phase arrest, caspase 9/3 activation, p53 up-regulation, and B-cell lymphoma 2 (Bcl-2) downregulation, all of which contribute to anticancer activities. ^12^ Bakuchiol has also shown inhibitory effects on DNA polymerase enzymes, while a related compound Bakuchicin inhibits the actions of topoisomerase II. ^65^ Its effects on DNA replication enzymes and relatively much lower cytotoxic effects than its analogue resveratrol make it a potent tolerable therapeutic compound for treating non-small cell lung cancer. ^12^

High doses of Bakuchiol (>2 μg/mL) inhibited cell proliferation of breast cancer MCF-7 cells through actions via estrogen receptors (ER), inducing ERβ expression and suppressing ERα expression. ^66^ S phase arrest was also observed in MCF-7 cells along with upregulation of ATM serine/threonine kinase (ATM), phosphorylated cell division cycle protein 2 (P-Cdc2), p21, myelin transcription factor 1 (Myt1), and downregulation of Cyclin B1, which implies the blocking of Cdc2 activation by upregulation of ERβ may play a role in the S phase arrest. ^66^ It also induced apoptosis in MCF-7 cells with an increase in expression of pro-apoptotic Bcl-2 and cleaved caspase proteins, pointing towards its involvement in apoptosis via the intrinsic mitochondrial pathway, similar to that observed in the lung adenocarcinoma A549 cell line and even liver cancer cell lines. ^12^^,^^66^

In addition, bakuchiol was found to effectively inhibit the activation of hypoxia-inducible factor 1 (HIF-1) and NFkB in gastric cancer AGS and cervical adenocarcinoma HeLa cell lines. ^67^ It also inhibits proliferation, migration and invasion of androgen-independent prostate cancer cell line PC-3 by inactivating NF-κB signaling via Androgen receptor (AR) and ERβ expression in a time and dose-dependent manner. Bakuchiol’s cytotoxicity was considered in this assay via LDH assay, and the non-toxic concentration used in further experimentation was determined to be at 10μM. ^68^

Bakuchiol-treated NUGC3 gastric cancer cells have also been shown to express reduced levels of phosphorylated protein kinase B (AKT) protein and increased p-extracellular signal-related kinase 1/2 (ERK1/2) and p-c-Jun N-terminal kinase (JNK) expression, indicating the induction of cell death was mitochondria-dependent, working via the MAPK/PI3K/AKT pathways. ^69^

Furthermore, bakuchiol was shown to induce estrogenic activity *in vivo* and *in vitro* study models. ^66^ It activates the ERβ receptor and suppresses the ERα receptor, which reduces CDC2 activity and promotes S phase arrest, reducing tumor cell proliferation. ^66^ This promotes bakuchiol as a phytoestrogen and anticancer drug, promoting safer hormone replacement therapeutics. Bakuchiol can also prevent bone loss and delay osteoporosis in post-menopausal women by activating the ERs. ^70^ This is of increased significance as hormone replacement therapies usually use estrogen, which is linked with an increased risk of breast cancer.

There have also been other compounds extracted from *Psoralea corylifolia L.*that express similar pharmacological activities. ^71^ Psoralidin, a natural phenolic coumarin, is one such compound that is beneficial in various diseases, including osteoporosis and hormonal cancers. ^72^ This is elucidated by inducing oxidative stress and apoptosis in tumorigenic cells, which promote autophagy-dependent cell death and activate the ER signaling pathway. The use of bakuchiol or other anticancer natural compounds should be evaluated to further check their efficacy in preventing or controlling cancer development. This would provide a suitable cheaper alternative to current cancer chemotherapeutics in the market, which are not accessible equitably among patients due to high costs.

***Antioxidative effects of bakuchiol***

There has been an increasing interest in identifying and characterizing antioxidant compounds to treat oxidative stress and related diseases, among which bakuchiol has been characterized as it has shown vital antioxidant activities in multiple studies. ^5^^,^^20^^,^^73^ When studying the scavenging activity of bakuchiol against various oxidizing radicals, it was determined that the terpenoid chains present in bakuchiol play a role in preventing lipid peroxidation. ^13^ It can protect against rat liver injury by inhibiting lipid peroxidation, ^74^ with similar effects expressed by studies on other natural products such as pumpkin seeds and acanthoic acid. ^75^ Bakuchiol can also inhibit liver fibrosis and show hepatoprotective effects by inhibiting oxidative stress while inducing apoptosis in myofibroblasts, relieving the hepatotoxicity of various toxicants. ^74^^,^^76^ Bakuchiol reduced cell death in retinal ganglion cells (RGC-5) and reduced ROS-induced apoptosis and cell death *in vitro.* ^49^

*In vivo* studies have demonstrated that bakuchiol reduces retinal degeneration following optic nerve injury. ^49^ It has also been observed that bakuchiol can protect against sepsis and sepsis-induced acute kidney injury by significantly reducing inflammation and renal oxidative stress while inhibiting induced activation of NFkB and p38-MAPK signaling pathways in the kidneys. ^77^ Its ability to block NFkB signaling also allows for cardioprotective functions, as seen in various mice models, allowing its application as a potential treatment for pathological cardiac hypertrophy. ^78^

In another study, bakuchiol showed protective effects against early brain injury by reducing ROS, malondialdehyde (MDA), 4-Hydroxynonenal (4-HNE), 3-Nitrotyrosine (3-NT), and other biomarkers of oxidative stress produced by lipid peroxidation. ^79^ Conversely, it causes increases in the enzyme activity of superoxide dismutase (SOD) and inducible glutathione peroxidase (GSH-Pxi), both of which play crucial roles in the body’s antioxidant defense system while reducing mitochondrial damage. As seen in this study, phosphorylation of AMP-activated protein kinase (AMPK) and thioredoxin 1 (Trx1) protein levels increased. In contrast, thioredoxin-interacting protein (TXNIP) levels decreased due to treatment with bakuchiol, which was thought to have occurred due to the regulation of Trx1 and TXNIP levels.

Bakuchiol may be considered a potential candidate for the prevention and treatment of insulin resistance as it can reduce induced insulin resistance and oxidative stress with reduced ROS expression and enhanced antioxidant enzyme expression. ^41^

Bakuchiol can also reduce the severity of myocardial ischemia-reperfusion injury (IRI) by impairing mitochondrial oxidative damage through regulation of sirtuin 1/proliferator-activated receptor gamma coactivator 1-alpha (SIRT1/PGC-1α) pathway signaling via increased in the expression of SIRT1. ^80^ It reduced mitochondrial oxidative damage by increasing the action of mitochondrial succinate dehydrogenase, cytochrome c oxidase, and mitochondrial SOD and decreased the production of malondialdehyde. ^79^

Bakuchiol has also increased anti-apoptotic Bcl2 and decreased pro-apoptotic Bax and cleaved caspase 3, which helps control injury-induced cell death, and inhibitors of SIRT1 abolished the effects of bakuchiol further highlight the role of its interaction with SIRT1 signaling. ^78^ Bakuchiol has also been found to reduce hyperglycemia-induced cardiomyopathy by activating the SIRT1/Nrf2 pathway, which reduces myocardial oxidative stress and elevates antioxidant production. ^73^

Similar effects are observed in other natural products such as curcumin, melatonin, berberine, and icariin. ^80^^–^^84^ These also reduce myocardial IRI via other pathways, highlighting the importance of studying the other potential signaling pathways regulated by bakuchiol, which leads to its ability to reduce injury-induced cell damage. Moreover, bakuchiol should be combined with other natural compounds with similar protective effects for potential synergistic activity.

***Antiviral effects of bakuchiol***

The antiviral effect of bakuchiol has been evaluated in some studies. ^15^^,^^85^^,^^86^ Viral diseases are constantly evolving in pathogenicity, and certain strains have been reported to be highly pathogenic to humans, as was seen in the influenza A pandemic in 1918, causing 50 million deaths, ^87^ and in the current pandemic caused by the SARS-CoV-2 virus, ^88^ which has infected more than 500 million people and has caused more than 6 million deaths since its first reported case on Dec. 31, 2019. ^89^ The SARS-CoV-2 virus enters the host cell via the interaction between its receptor-binding domain (RBD) of spike glycoprotein with the angiotensin-converting enzyme 2 (ACE2) receptor found on the plasma membrane of the host cell. ^90^ Given the rapid emergence of resistant viral strains, searching for potent and tolerable antiviral drugs is essential to combat and control viral infections.

Plant-based natural compounds are being examined extensively for their therapeutic effects, ^91^^,^^92^ and bakuchiol has also been evaluated, given its use in traditional medicine systems to treat a wide range of diseases. In *in vitro* studies on Madin-Darby canine kidney (MDCK) cells infected with influenza A H1N1 strain, the naturally occurring form of bakuchiol was found to inhibit influenza A growth and infection while reducing expression of viral mRNAs and proteins, decreasing viral load. ^15^ In addition, it also was able to activate Nrf2 and two Nrf2-induced genes, NAD(P)H quinone oxidoreductase 1 and glutathione S-transferase A3, promoting activation of transcriptional regulation and regulating virus-induced host body oxidative stress response. To a lesser extent, these effects were also observed in its enantiomer form, highlighting the importance of chirality in designing potent antiviral drugs.

Further studies on related compounds such as cyclobakuchiols A, B, and C, derived from (+)-(S)-Bakuchiol as well as from its natural sources, have also established strong potential to inhibit viral growth, infection, and expression of viral mRNAs and proteins in influenza A virus-infected MDCK cells via similar mechanisms. ^85^ Other natural compounds derived from natural sources show antiviral actions against Influenza A, such as aurantiamide acetate extracted from the plant’s roots *Baphicacanthus cusia.* ^37^ These compounds work via similar mechanisms as bakuchiol, as observed in MDCK cells. In addition, these natural compounds also reduce virus-induced inflammatory responses via the suppression of NF-kB signaling pathways and pro-inflammatory cytokines. ^93^

In a more recent study, bakuchiol effectively inhibited severe acute respiratory syndrome coronavirus 2 (SARS-CoV-2) pseudovirus entry at concentrations of up to 100μM without toxicity. Furthermore, in HEK293 cell lines overexpressing human ACE2 receptors, bakuchiol also effectively blocked RBD-ACE2 binding at the cell membrane. ^86^ This also implies that further evaluation of bakuchiol on its effect on other viral diseases is critical in the current pandemic setting caused by the SARS-CoV-2 virus to find effective antiviral treatments to reduce disease transmission and severity.

Other natural compounds have also been evaluated in recent studies, among which epigallocatechin gallate (EGCG), 20(S)-ginsenoside Rg3 (SRg3), 20(R)-ginsenoside Rg3 (RRg3), isobavachalcone (Ibvc), and isochlorogenic A (IscA) were found to effectively inhibited pseudovirus entry at concentrations up to 100 μM. ^86^^,^^94^

Among these compounds, EGCG and lbvc have shown comparable effects to bakuchiol administration by inhibiting the SARS-CoV-2-induced cytopathic effect and plaque formation and demonstrating a dual binding to RBD and ACE2. ^93^ Another compound, 1,2,3,4,6-O-Pentagalloylglucose (PGG), also effectively inhibited virus binding and infection in ACE2 overexpressing human host cells, binding more with Spike-RBD than ACE2 receptors. ^95^ It blocks the fusion of SARS-CoV-2 to hACE2 receptors on a dose-dependent level. Spike RBD PGG was also found to exhibit anti-influenza-virus activity by reducing the accumulation of nucleoprotein and viral hemagglutinin in plasma membranes at the late stage of the replication cycle and inhibiting the release of progeny virus from infected cells. PGG also works in ameliorating HBV, HCV, HIV infections. ^95^ PGG may be a safe and potential antiviral agent against COVID-19 by blocking the fusion of SARS-CoV-2 spike-RBD.

***Combination effects***

Most studies evaluating the effects of bakuchiol used in combination with other molecules tend to be formulated by the cosmeceutical industry. It is also used in combination with retinol to help increase its photostability, or moisturizing molecules like squalene, working synergistically to improve skin elasticity and texture.

Bakuchiol can be combined with *Vanilla tahitensis* extract to inhibit skin photoaging and cause improvement in skin barrier function and elasticity, increasing cellular turnover and improving signs of aging. ^10^ A study by Bacqueville *et al*. used an *in vitro* skin model made of human dermal fibroblasts treated with the compounds alone or in combination, after which they were exposed to an acute dose of UVA. Exposure to UVA-induced significant morphological changes and increased IL-8 and p16 expression in the control model (no treatment), suggesting inflammation and senescence. Compared to control, treatment with either compound alone prevented actin network alteration and IL-8 upregulation, while protecting against IL-8 and p16 overexpression in combination. This combination was also formulated into serum and tested in participants who applied it twice daily for 56 days. These compounds can work synergistically to reduce ptosis and skin deformation and improve the radiance of naturally aged skin in women.

A face serum containing bakuchiol, palmitoyl tripeptide-38, hydrolyzed hyaluronic acid and a polyherbal and vitamin blend was tested in 55 healthy adults. Daily use for 3 months indicated improvements in skin as seen in *in vitro* studies and clinical trials on healthy volunteers. ^60^ Protection of skin structure was observed *in vitro* with reduced collagenase activity and significant free radical scavenging activity as observed through increased gene expression of dermal collagen, elastin and hyaluronic acid synthesis. In addition, substantial improvements in skin elasticity, hydration, roughness (fine lines and wrinkles), and brightness occurred during the trial.

It also works marvelously in combination with melatonin and ascorbyl tetraisopalmitate to cause significant clinical anti-aging effects when applied once daily, ^96^ with a statistically significant decrease in wrinkles and redness, an increase in skin firmness and overall improvement in skin quality and complexion as well as hydration. ^96^ A combination of bakuchiol, Ginkgo biloba extract and mannitol was also shown to improve the efficacy of adapalene treatment in patients suffering from Acne Vulgaris. ^9^ It has also been found to work exceptionally well with salicylic acid in managing P. acnes. ^97^

Outside of the dermatocosmetic industry, the use of bakuchiol can also improve the efficacy of various non-cosmetic treatments. Bakuchiol has been found to work in combination with tumor necrosis factor (TNF)- related apoptosis-inducing ligand (TRAIL) to inhibit the growth of TRAIL sensitive (HCT116) and resistant (HT-29) colon cancer cell lines. ^98^ Combination treatment of bakuchiol with TRAIL on these cell lines significantly upregulated the expression of TRAIL cell death receptors DR4 and DR5 in a dose-dependent manner, as well as the expression of the pro-apoptotic proteins PARP and the cleaved caspases 3, 8 and 9, while suppressing the expression of survival proteins such as cFLIP, survivin, XIAP and Bcl2. Pretreatment of cells with JNK inhibitor SP600125 and ROS scavenger N-acetylcysteine and the depletion of DR4 or DR5 by small interfering RNA reduced the bakuchiol-induced cell growth inhibition Bakuchiol assists with TRAIL-induced apoptosis via the ROS/JNK pathway.

When used alone or in combination with A *llium sativum*, it also demonstrated potent antimicrobial properties, with bakuchiol and A *llium sativum* showing synergistic effects when used in combination. ^99^

### Side effects

Bakuchiol is generally used in the cosmeceutical field as a more tolerable version of retinol, having retinol functionality through retinol-like regulation of gene expression. However, it may initially cause some redness and peeling in sensitive skin, ^4^ although chances are rare due to the established anti-inflammatory nature of bakuchiol. As it may increase cellular turnover, sunscreen is recommended for use after applying bakuchiol-containing products to reduce damage by UV radiation. Retinol and its derivatives are generally discouraged for use during pregnancy. No studies have been done to evaluate use safety in pregnant women, so use should be carefully conducted. It is difficult to quantify the benefits and side effects of bakuchiol usage as most studies have been done *in vitro*, potentially introducing a risk of bias.

It should be mentioned that studies have found bakuchiol to be non-toxic to cell cultures even in high concentrations of up to 5000 uG/mL, ^100^ however, this was only observed in *in vitro* studies, and further studies are required to determine dosage toxicity. Recent clinical reports have indicated that treatment with *Psoraleae Fructus (PF),* an essential source of Bakuchiol, is associated with an increased risk of liver injury. ^25^^,^^26^^,^^100^^–^^104^ A study by Guo *et al.* (2021) indicated that bakuchiol, among other constituents of *Psoraleae Fructus* induced oxidative stress and mitochondrial damage-mediated apoptosis, alleviated when Ethanol extracts of PF were used. Bakuchiol may induce cholestatic hepatotoxicity as treatment with Bakuchiol reduces mRNA expression of CYP7A1, HMG-CoA reductase, PPARα, and SREBP-2. ^105^

Care should be taken when using bakuchiol in combination therapy because it may cause an increase in cytotoxic effects of bakuchiol, and extensive studies should be conducted to ensure that metabolic toxicity does not occur. Bakuchiol can induce nephrotoxicity when it is used in combination with other natural ingredients such as Glycyrrhetinic acid (GA) found in licorice, which inhibits the CYP450 isoenzymes (CYP3A4, CYP2C9, CYP1A2) involved in metabolic detoxification of bakuchiol. ^106^ The presence of GA altered the toxicokinetics of bakuchiol in rats, increased the internal exposure, suppressed the elimination of the bakuchiol prototype, and therefore may have enhanced the renal nephrotoxicity.

## Conclusions

This review focused on the pharmacological benefits of the compound bakuchiol, traditionally isolated from the bakuchi plant and used in traditional medicine for centuries. Recent studies have highlighted its vital role in controlling several activities that lead to health depreciation and the onset of various non-communicable diseases. Additionally, bakuchiol has shown potent antimicrobial and antiviral responses against various pathogens in multiple studies, which allows for the development of potential novel cures and preventive strategies.

Moreover, extensive research is required on the non-toxic extraction of the compounds, fully realizing its pharmacological and biochemical modes of action, and planning for sustainable ways of growing its sources to meet the increasing demands of the pharmaceutical industries. Further studies should also be done to evaluate the long-term effects of prolonged bakuchiol consumption, possibly via retrospective analyses, which have not been done yet.

## Data Availability

No data are associated with this article.

## References

[1] Improving access to essential medicines: World Health Organization. 2007 [cited 2022 Jun 3]. Reference Source

[2] Wyk AS van Prinsloo G : Medicinal plant harvesting, sustainability and cultivation in South Africa. *Biol. Conserv.* 2018 Nov;227:335–342.

[3] Subramani R Lakshmanaswamy R : Complementary and Alternative Medicine and Breast Cancer. *Prog. Mol. Biol. Transl. Sci.* 2017 Jan 1;151:231–274. 10.1016/bs.pmbts.2017.07.008 29096896

[4] Chaudhuri RK Bojanowski K : Bakuchiol: a retinol-like functional compound revealed by gene expression profiling and clinically proven to have anti-aging effects. *Int. J. Cosmet. Sci.* 2014;36(3):221–230. 10.1111/ics.12117 24471735

[5] Xin Z Wu X Ji T : Bakuchiol: A newly discovered warrior against organ damage. *Pharmacol. Res.* 2019 Mar;141:208–213. 10.1016/j.phrs.2019.01.001 30610961

[6] Choi SY Lee S Choi WH : Isolation and anti-inflammatory activity of bakuchiol from ulmus davidiana var. japonica. *J. Med. Food.* 2010 Aug 1 [cited 2021 Nov 9];13(4):1019–1023. 10.1089/jmf.2009.1207 20553183

[7] Lim HS Kim YJ Kim BY : Bakuchiol Suppresses Inflammatory Responses Via the Downregulation of the p38 MAPK/ERK Signaling Pathway. *Int. J. Mol. Sci.* 2019 Jul;20(14):3574. 10.3390/ijms20143574 31336605 PMC6678636

[8] Pae HO Cho H Oh GS : Bakuchiol from Psoralea corylifolia inhibits the expression of inducible nitric oxide synthase gene via the inactivation of nuclear transcription factor-κB in RAW 264.7 macrophages. *Int. Immunopharmacol.* 2001 Sep;1(9–10):1849–1855. 10.1016/S1567-5769(01)00110-2 11562076

[9] Poláková K Fauger A Sayag M : A dermocosmetic containing bakuchiol, Ginkgo biloba extract and mannitol improves the efficacy of adapalene in patients with acne vulgaris: result from a controlled randomized trial. *Clin. Cosmet. Investig. Dermatol.* 2015 Apr;8:187–191. 10.2147/CCID.S81691 25914553 PMC4401329

[10] Bacqueville D Maret A Noizet M : Efficacy of a Dermocosmetic Serum Combining Bakuchiol and Vanilla Tahitensis Extract to Prevent Skin Photoaging in vitro and to Improve Clinical Outcomes for Naturally Aged Skin. *Clin. Cosmet. Investig. Dermatol.* 2020;13:359–370. 10.2147/CCID.S235880 32494181 PMC7231787

[11] Katsura H Tsukiyama RI Suzuki A : In vitro antimicrobial activities of bakuchiol against oral microorganisms. *Antimicrob. Agents Chemother.* 2001;45(11):3009–3013. 10.1128/AAC.45.11.3009-3013.2001 11600349 PMC90775

[12] Chen Z Jin K Gao L : Anti-tumor effects of bakuchiol, an analogue of resveratrol, on human lung adenocarcinoma A549 cell line. *Eur. J. Pharmacol.* 2010 Sep;643(2–3):170–179. 10.1016/j.ejphar.2010.06.025 20599920

[13] Haraguchi H Inoue J Tamura Y : Inhibition of mitochondrial lipid peroxidation by Bakuchiol, a meroterpene from Psoralea corylifolia. *Planta Med.* 2000;66(6):569–571. 10.1055/s-2000-8605 10985089

[14] Khuranna D Sharma S Mir SR : Extraction, Quantification, and Cytokine Inhibitory Response of Bakuchiol in Psoralea coryfolia Linn. *Separations.* 2020 Sep;7(3):48. 10.3390/separations7030048 Reference Source

[15] Shoji M Arakaki Y Esumi T : Bakuchiol is a phenolic isoprenoid with novel enantiomer-selective anti-influenza a virus activity involving Nrf2 activation. *J. Biol. Chem.* 2015 Nov 13 [cited 2021 Nov 9];290(46):28001–28017. 10.1074/jbc.M115.669465 Reference Source 26446794 PMC4646038

[16] Mehta G Nayak UR Dev S : Bakuchiol, a novel monoterpenoid. *Tetrahedron Lett.* 1966 Jan;7(38):4561–4567. 10.1016/S0040-4039(00)70078-5

[17] Mahajan N Koul B Gupta P : Psoralea corylifolia L.: Panacea to several maladies. *South African J. Bot.* 2022 Sep 1;149:963–993. 10.1016/j.sajb.2022.01.024

[18] Ohno O Watabe T Nakamura K : Inhibitory effects of bakuchiol, bavachin, and isobavachalcone isolated from Piper longum on melanin production in B16 mouse melanoma cells. *Biosci. Biotechnol. Biochem.* 2010;74(7):1504–1506. 10.1271/bbb.100221 20622433

[19] Krenisky JM Luo J Reed MJ : Isolation and antihyperglycemic activity of bakuchiol from Otholobium pubescens (Fabaceae), a Peruvian medicinal plant used for the treatment of diabetes. *Biol. Pharm. Bull.* 1999;22(10):1137–1140. 10.1248/bpb.22.1137 10549873

[20] Adarsh Krishna TP Edachery B Athalathil S : Bakuchiol - a natural meroterpenoid: structure, isolation, synthesis and functionalization approaches. 2022 Mar;12(14):8815–8832. 10.1039/D1RA08771A PMC8985110 35424800

[21] Sofowora A Ogunbodede E Onayade A : The Role and Place of Medicinal Plants in the Strategies for Disease Prevention. *Afr. J. Tradit. Complement. Altern. Med.* 2013;10(5):229. 10.4314/ajtcam.v10i5.2 PMC3847409 24311829

[22] Khushboo P Jadhav V Kadam V Sathe N : Psoralea corylifolia Linn.—“Kushtanashini.” *Pharmacogn. Rev.* 2010 Jan;4(7):69. 10.4103/0973-7847.65331 22228944 PMC3249905

[23] Niu C Lu X Aisa HA : Preparation of novel 1,2,3-triazole furocoumarin derivatives via click chemistry and their anti-vitiligo activity. *RSC Adv.* 2019 Jan 9 [cited 2022 Dec 6];9(3):1671–1678. 10.1039/C8RA09755K Reference Source 35518056 PMC9059643

[24] Stéphane FFY Jules BKJ Batiha GES : Extraction of Bioactive Compounds from Medicinal Plants and Herbs. *Natural Medicinal Plants. IntechOpen.* 2021. undefined/state.item.id.

[25] Nam SW Baek JT Lee DS : A case of acute cholestatic hepatitis associated with the seeds of Psoralea corylifolia (Boh-Gol-Zhee). *Clin. Toxicol. (Phila.).* 2005;43(6):589–591. 10.1081/CLT-200068863 16255343

[26] Li YJ Huang YY : Drug-induced liver injury caused by Psoralea corylifolia: a case report. *Shanghai Med Pharm.* 2016;97(24):41–42.

[27] Zhang QW Lin LG Ye WC : Techniques for extraction and isolation of natural products: A comprehensive review. *Chinese Med (United Kingdom).* 2018 Apr;13(1):20–26. 10.1186/s13020-018-0177-x 29692864 PMC5905184

[28] Li P Xu G Li SP : Optimizing ultraperformance liquid chromatographic analysis of 10 diterpenoid compounds in Salvia miltiorrhiza using central composite design. *J. Agric. Food Chem.* 2008 Feb 27 [cited 2021 Dec 31];56(4):1164–1171. 10.1021/jf073020u 18198831

[29] Li P Yin ZQ Li SL : Simultaneous determination of eight flavonoids and pogostone in pogostemon cablin by high performance liquid chromatography. 2014 Jul 21 [cited 2021 Dec 31];37(12):1771–84. 10.1080/10826076.2013.809545

[30] Esumi T Yamamoto C Fukuyama Y : A short synthesis of (+)-bakuchiol. *Synlett.* 2013;24(14):1845–1847. 10.1055/s-0033-1338968

[31] Li CC Wang TL Zhang ZQ : Phytochemical and Pharmacological Studies on the Genus Psoralea: A Mini Review. *Evidence-based Complement Altern Med.* 2016;2016:1–17. 10.1155/2016/8108643 27956922 PMC5124476

[32] Cho YH Ahn GW Yang SW : Development of Bioavailability Enhancement System for the Skin Permeation Promotion of Psolarea corylifolia Extract. *KSBB J.* 2011 Dec;26(6):505–512. 10.7841/ksbbj.2011.26.6.505

[33] Kibe MN Konyole S Kathure D : The role of phytochemicals in prevention and control of chronic diseases. *Int. J. Curr. Res.* 2017;9(12):62540–62543.

[34] Kany S Vollrath JT Relja B : Cytokines in Inflammatory Disease. *Int. J. Mol. Sci.* 2019 Dec 1 [cited 2021 Nov 9];20(23). 10.3390/ijms20236008 31795299 PMC6929211

[35] Ferrándiz ML Gil B Sanz MJ : Effect of bakuchiol on leukocyte functions and some inflammatory responses in mice. *J. Pharm. Pharmacol.* 1996;48(9):975–980. 10.1111/j.2042-7158.1996.tb06016.x 8910867

[36] Lee SW Yun BR Kim MH : Phenolic compounds isolated from Psoralea corylifolia inhibit IL-6-induced STAT3 activation. *Planta Med.* 2012;78(9):903–906. 10.1055/s-0031-1298482 22573369

[37] Zhou B Yang Z Feng Q : Aurantiamide acetate from baphicacanthus cusia root exhibits anti-inflammatory and anti-viral effects via inhibition of the NF-$κ$B signaling pathway in Influenza A virus-infected cells. *J. Ethnopharmacol.* 2017 Mar;199:60–67. 10.1016/j.jep.2017.01.038 28119097

[38] Brandt SL Serezani CH : Too much of a good thing: How modulating LTB4 actions restore host defense in homeostasis or disease. *Semin. Immunol.* 2017 Oct;33:37–43. 10.1016/j.smim.2017.08.006 29042027 PMC5679129

[39] Woo CH You HJ Cho SH : Leukotriene B(4) stimulates Rac-ERK cascade to generate reactive oxygen species that mediates chemotaxis. *J. Biol. Chem.* 2002 Mar;277(10):8572–8578. 10.1074/jbc.M104766200 11756405

[40] Xu Q Lv Q Liu L : New bakuchiol dimers from Psoraleae Fructus and their inhibitory activities on nitric oxide production. *Chin. Med.* 2021 Dec;16(1):98. 10.1186/s13020-021-00499-y 34620201 PMC8499495

[41] Truong CS Seo E Jun HS : Psoralea corylifolia L. Seed Extract Attenuates Methylglyoxal-Induced Insulin Resistance by Inhibition of Advanced Glycation End Product Formation. *Oxidative Med. Cell. Longev.* 2019;2019:1–14. 10.1155/2019/4310319 PMC6954480 31976027

[42] Kim YJ Lim HS Lee J : Quantitative Analysis of Psoralea corylifolia Linne and its Neuroprotective and Anti-Neuroinflammatory Effects in HT22 Hippocampal Cells and BV-2 Microglia. *Molecules.* 2016 Aug;21(8):1076. 10.3390/molecules21081076 27548120 PMC6274380

[43] Zhou P Hua F Wang X : Therapeutic potential of IKK-β inhibitors from natural phenolics for inflammation in cardiovascular diseases. *Inflammopharmacology.* 2020 Feb;28(1):19–37. 10.1007/s10787-019-00680-8 31894515

[44] Holler JG Christensen SB Slotved HC : Novel inhibitory activity of the Staphylococcus aureus NorA efflux pump by a kaempferol rhamnoside isolated from Persea lingue Nees. *J. Antimicrob. Chemother.* 2012 May;67(5):1138–1144. 10.1093/jac/dks005 Reference Source 22311936

[45] Nawrot R Barylski J Nowicki G : Plant antimicrobial peptides. *Folia Microbiol. (Praha).* 2014 Oct;59(3):181–196. 10.1007/s12223-013-0280-4 24092498 PMC3971460

[46] Mahizan NA Yang SK Moo CL : Terpene Derivatives as a Potential Agent against Antimicrobial Resistance (AMR) Pathogens. *Molecules.* 2019 Jul;24(14):2631. 10.3390/molecules24142631 Reference Source 31330955 PMC6680751

[47] Hsu PJ Miller JS Berger JM : Bakuchiol, an antibacterial component of Psoralidium tenuiflorum. *Nat. Prod. Res.* 2009;23(8):781–788. 10.1080/14786410902840158 19418361

[48] Vahabi S Najafi E Alizadeh S : In vitro antimicrobial effects of some herbal essences against oral pathogens. *J. Med. Plant Res.* 2011 Sep;5(19):4870–4878. Reference Source

[49] Kim KA Shim SH Ahn HR : Protective effects of the compounds isolated from the seed of Psoralea corylifolia on oxidative stress-induced retinal damage. *Toxicol. Appl. Pharmacol.* 2013 Jun;269(2):109–120. 10.1016/j.taap.2013.03.017 23545180

[50] Stan D Enciu AM Mateescu AL : Natural Compounds With Antimicrobial and Antiviral Effect and Nanocarriers Used for Their Transportation. *Front. Pharmacol.* 2021 Sep;12. 10.3389/fphar.2021.723233 34552489 PMC8450524

[51] Cowan MM : Plant Products as Antimicrobial Agents. *Clin. Microbiol. Rev.* 1999;12(4):582. 10.1128/cmr.12.4.564 PMC88925 10515903

[52] Khameneh B Iranshahy M Soheili V : Review on plant antimicrobials: a mechanistic viewpoint. Antimicrob Resist. *Infect. Control.* 2019 Jul;8(1). 10.1186/s13756-019-0559-6 Reference Source/ PMC6636059 31346459

[53] Gonelimali FD Lin J Miao W : Antimicrobial Properties and Mechanism of Action of Some Plant Extracts Against Food Pathogens and Spoilage Microorganisms. *Front. Microbiol.* 2018 Jul;9(JUL):1639. 10.3389/fmicb.2018.01639 , 30087662 PMC6066648

[54] Cox J : Antimicrobial resistance now causes more deaths than HIV/AIDS and malaria worldwide – new study *Vaccines Work.* 2022 [cited 2022 Jun 3]. Reference Source

[55] Cunningham A : Antimicrobial resistance is a leading cause of death globally. *Sci. News.* 2022 [cited 2022 Jun 3]; Reference Source

[56] Mallepally VR Thota N Payare LS : Novel bisstyryl derivatives of bakuchiol: targeting oral cavity pathogens. *Eur. J. Med. Chem.* 2010;45(7):3125–3134. 10.1016/j.ejmech.2010.03.049 Reference Source 20427099

[57] Lau KM Fu LH Cheng L : Two antifungal components isolated from Fructus Psoraleae and Folium Eucalypti Globuli by bioassay-guided purification. *Am. J. Chin. Med.* 2010;38(5):1005–1014. 10.1142/S0192415X10008421 20821830

[58] Nordin MAF Abdul Razak F Himratul-Aznita WH : Assessment of Antifungal Activity of Bakuchiol on Oral-Associated Candida spp. *Evid. Based Complement. Alternat. Med.* 2015;2015:1–7. 10.1155/2015/918624 26633986 PMC4655055

[59] Dhaliwal S Rybak I Ellis SR : Prospective, randomized, double-blind assessment of topical bakuchiol and retinol for facial photoageing. *Br. J. Dermatol.* 2019 Feb;180(2):289–296. 10.1111/bjd.16918 29947134

[60] West BJ Alabi I Deng S : A Face Serum Containing Bakuchiol, Palmitoyl Tripeptide-38, Hydrolyzed Hyaluronic Acid and a Polyherbal and Vitamin Blend Improves Skin Quality in Human Volunteers and Protects Skin Structure In vitro. *Preprints.* 2021 Jun. Reference Source1

[61] Yu Q Zou HM Wang S : Regulative effect of bakuchiol on ESF-1 cells anti-aging gene. *J. Chinese Med. Mater.* 2014 Apr;37(4):632–635. 25345139

[62] Draelos ZD Gunt H Zeichner J : Clinical Evaluation of a Nature-Based Bakuchiol Anti-Aging Moisturizer for Sensitive Skin. *J Drugs Dermatol.* 2020 Dec;19(12):1181–1183. 10.36849/JDD.2020.5522 Reference Source 33346506

[63] Woolery-Lloyd HC Keri J Doig S : Retinoids and Azelaic Acid to Treat Acne and Hyperpigmentation in Skin of Color. *J. Drugs Dermatol.* 2013 Apr;12(4):434–437. Reference Source 23652891

[64] Gimeno A Zaragozá R Vivó-Sesé I : Retinol, at concentrations greater than the physiological limit, induces oxidative stress and apoptosis in human dermal fibroblasts. *Exp. Dermatol.* 2004 Jan;13(1):45–54. 10.1111/j.0906-6705.2004.00112.x 15009115

[65] Sun NJ Woo SH Cassady JM : DNA polymerase and topoisomerase II inhibitors from Psoralea corylifolia. *J. Nat. Prod.* 1998 Mar;61(3):362–366. 10.1021/np970488q Reference Source 9544566

[66] Li L Chen X Liu CC : Phytoestrogen Bakuchiol Exhibits in vitro and in vivo Anti-breast Cancer Effects by Inducing S Phase Arrest and Apoptosis. *Front. Pharmacol.* 2016;7(MAY):128. /pmc/articles/PMC4877368. 10.3389/fphar.2016.00128 27252650 PMC4877368

[67] Majeed R Reddy MV Chinthakindi PK : Bakuchiol derivatives as novel and potent cytotoxic agents: A report. *Eur. J. Med. Chem.* 2012 Mar 1;49:55–67. 10.1016/j.ejmech.2011.12.018 22245048

[68] Miao L Yun X Tao R : Bakuchiol exhibits anti-metastasis activity through NF-κB cross-talk signaling with AR and ERβ in androgen-independent prostate cancer cells PC-3. *J. Pharmacol. Sci.* 2018 Sep;138(1):1–8. 10.1016/j.jphs.2017.04.004 30236540

[69] Lv L Liu B : Anti-tumor effects of bakuchiol on human gastric carcinoma cell lines are mediated through PI3K/AKT and MAPK signaling pathways. *Mol. Med. Rep.* 2017 Dec;16(6):8977–8982. 10.3892/mmr.2017.7696 28990045

[70] Lim SH Ha TY Kim SR : Ethanol extract of *Psoralea corylifolia* L. and its main constituent, bakuchiol, reduce bone loss in ovariectomised Sprague-Dawley rats. *Br. J. Nutr.* 2009;101(7):1031–1039. 10.1017/S0007114508066750 18801207

[71] Alam F Khan GN Asad MHH : Bin. *Psoralea corylifolia* L: Ethnobotanical, biological, and chemical aspects: A review. *Phyther Res.* 2018 Apr;32(4):597–615. 10.1002/ptr.6006 29243333 PMC7167735

[72] Xin Z Wu X Yu Z : Mechanisms explaining the efficacy of psoralidin in cancer and osteoporosis, a review. *Pharmacol. Res.* 2019 Sep;147:104334. 10.1016/j.phrs.2019.104334 31255708

[73] Ma W Guo W Shang F : Bakuchiol Alleviates Hyperglycemia-Induced Diabetic Cardiomyopathy by Reducing Myocardial Oxidative Stress via Activating the SIRT1/Nrf2 Signaling Pathway. *Oxidative Med. Cell. Longev.* 2020;2020:1–15. 10.1155/2020/3732718 PMC7545423 33062139

[74] Park EJ Zhao YZ Kim YC : Protective effect of (S)-bakuchiol from Psoralea corylifolia on rat liver injury in vitro and in vivo. *Planta Med.* 2005 Jun;71(6):508–513. 10.1055/s-2005-864150 15971120

[75] Park EJ Zhao YZ Kim YH : Acanthoic acid from Acanthopanax koreanum protects against liver injury induced by tert-butyl hydroperoxide or carbon tetrachloride in vitro and in vivo. *Planta Med.* 2004 Apr;70(4):321–327. 10.1055/s-2004-818943 15095147

[76] Park EJ Zhao YZ Kim YC : Bakuchiol-induced caspase-3-dependent apoptosis occurs through c-Jun NH2-terminal kinase-mediated mitochondrial translocation of Bax in rat liver myofibroblasts. *Eur. J. Pharmacol.* 2007 Mar;559(2–3):115–123. 10.1016/j.ejphar.2007.01.024 17292878

[77] Wang J Luo M Shen J : Bakuchiol from Psoralea corylifolia L. Ameliorates acute kidney injury and improves survival in experimental polymicrobial sepsis. *Int. Immunopharmacol.* 2020 Dec;89(Pt A):107000. 10.1016/j.intimp.2020.107000 Reference Source 33039956

[78] Wang Z Gao L Xiao L : Bakuchiol protects against pathological cardiac hypertrophy by blocking NF-$κ$B signaling pathway. *Biosci. Rep.* 2018 Oct;38(5). 10.1042/BSR20181043 Reference Source PMC6209581 30242058

[79] Liu H Guo W Guo H : Bakuchiol Attenuates Oxidative Stress and Neuron Damage by Regulating Trx1/TXNIP and the Phosphorylation of AMPK After Subarachnoid Hemorrhage in Mice. *Front. Pharmacol.* 2020 May;11:712. 10.3389/fphar.2020.00712 32499702 PMC7243250

[80] Feng J Yang Y Zhou Y : Bakuchiol attenuates myocardial ischemia reperfusion injury by maintaining mitochondrial function: the role of silent information regulator 1. *Apoptosis.* 2016 May;21(5):532–545. 10.1007/s10495-016-1225-6 27000151

[81] Yang Y Duan W Lin Y : SIRT1 activation by curcumin pretreatment attenuates mitochondrial oxidative damage induced by myocardial ischemia reperfusion injury. *Free Radic. Biol. Med.* 2013;65:667–679. 10.1016/j.freeradbiomed.2013.07.007 23880291

[82] Yang Y Duan W Jin Z : JAK2/STAT3 activation by melatonin attenuates the mitochondrial oxidative damage induced by myocardial ischemia/reperfusion injury. *J. Pineal Res.* 2013 Oct;55(3):275–286. 10.1111/jpi.12070 Reference Source 23796350

[83] Yu L Li Q Yu B : Berberine Attenuates Myocardial Ischemia/Reperfusion Injury by Reducing Oxidative Stress and Inflammation Response: Role of Silent Information Regulator 1. *Oxidative Med. Cell. Longev.* 2016;2016. Reference Source 10.1155/2016/1689602 PMC4691633 26788242

[84] Wu B Yu Feng J Ming Yu L : Icariin protects cardiomyocytes against ischaemia/reperfusion injury by attenuating sirtuin 1-dependent mitochondrial oxidative damage. *Br. J. Pharmacol.* 2018 Nov;175(21):4137–4153. 10.1111/bph.14457 Reference Source 30051466 PMC6177614

[85] Shoji M Esumi T Tanaka N : Organic synthesis and anti-influenza A virus activity of cyclobakuchiols A, B, C, and D. *PLoS One.* 2021 Mar;16(3):e0248960. 10.1371/journal.pone.0248960 Reference Source 33770117 PMC7997032

[86] Zhang D Hamdoun S Chen R : Identification of natural compounds as SARS-CoV-2 entry inhibitors by molecular docking-based virtual screening with bio-layer interferometry. *Pharmacol. Res.* 2021 Oct;172:105820. 10.1016/j.phrs.2021.105820 34403732 PMC8364251

[87] Taubenberger JK Morens DM : 1918 Influenza: the Mother of All Pandemics. *Emerg. Infect. Dis.* 2006 Jan;12(1):15–22. 10.3201/eid1209.05-0979 16494711 PMC3291398

[88] Hossain A Nasrullah SM Tasnim Z : Seroprevalence of SARS-CoV-2 IgG antibodies among health care workers prior to vaccine administration in Europe, the USA and East Asia: a systematic review and meta-analysis. *EClinicalMedicine.* 2021 Mar;33:100770. 10.1016/j.eclinm.2021.100770 33718853 PMC7938754

[89] COVID Live Update: 287,054,304 Cases and 5,449,037 Deaths from the Coronavirus - Worldometer. 2021.

[90] Davidson AM Wysocki J Batlle D : Interaction of SARS-CoV-2 and Other Coronavirus With ACE (Angiotensin-Converting Enzyme)-2 as Their Main Receptor: Therapeutic Implications. *Hypertension.* 2020 Sep;76:1339–1349. 10.1161/HYPERTENSIONAHA.120.15256 32851855 PMC7480804

[91] Ali SI Sheikh WM Rather MA : Medicinal plants: Treasure for antiviral drug discovery. *Phyther. Res.* 2021 Jul;35(7):3447–3483. 10.1002/ptr.7039 33590931 PMC8013762

[92] Bhuiyan FR Howlader S Raihan T : Plants Metabolites: Possibility of Natural Therapeutics Against the COVID-19 Pandemic. *Front. Med.* 2020 Aug;7. 10.3389/fmed.2020.00444 32850918 PMC7427128

[93] Al-Salihi SAA Alberti F : Naturally Occurring Terpenes: A Promising Class of Organic Molecules to Address Influenza Pandemics. *Nat. Products Bioprospect.* 2021 Aug;11(4):405–419. 10.1007/s13659-021-00306-z PMC8090910 33939136

[94] Mani JS Johnson JB Steel JC : Natural product-derived phytochemicals as potential agents against coronaviruses: A review. *Virus Res.* 2020 Jul;284:197989. 10.1016/j.virusres.2020.197989 32360300 PMC7190535

[95] Chen RH Yang LJ Hamdoun S : 1,2,3,4,6-Pentagalloyl Glucose, a RBD-ACE2 Binding Inhibitor to Prevent SARS-CoV-2 Infection. *Front. Pharmacol.* 2021 Mar;12:634176. 10.3389/fphar.2021.634176 33897423 PMC8058605

[96] Goldberg DJ Robinson DM Granger C : Clinical evidence of the efficacy and safety of a new 3-in-1 anti-aging topical night serum-in-oil containing melatonin, bakuchiol, and ascorbyl tetraisopalmitate: 103 females treated from 28 to 84 days. *J. Cosmet. Dermatol.* 2019 Jun;18(3):806–814. 10.1111/jocd.12896 30924254

[97] Chaudhuri RK Marchio F : Bakuchiol in the Management of Acne-affected Skin. *Cosmet Toilet.* 2011 Jul;126:502–510.

[98] Park MH Kim JH Chung YH : Bakuchiol sensitizes cancer cells to TRAIL through ROS- and JNK-mediated upregulation of death receptors and downregulation of survival proteins. *Biochem. Biophys. Res. Commun.* 2016 Apr;473(2):586–592. 10.1016/j.bbrc.2016.03.127 27033605

[99] Singh B Sharma RA : Plant terpenes: defense responses, phylogenetic analysis, regulation and clinical applications. 3. *Biotech.* 2015 Apr;5(2):129–151. 10.1007/s13205-014-0220-2 28324581 PMC4362742

[100] Li YF Cheng GF : Pharmaceutical Care for Patients with Liver Damage Induced by Traditional Chinese Medicine Fructus Psoraleae by Clinical Pharmacists. *Eval. Anal. Drug-Use Hosp. Chin.* 2018;16(4):571–573.

[101] Teschke R Bahre R : Severe hepatotoxicity by Indian Ayurvedic herbal products: A structured causality assessment. *Ann. Hepatol.* 2009 Jul;8(3):258–266. 10.1016/S1665-2681(19)31777-6 19841509

[102] Smith DA MacDonald S : A rare case of acute hepatitis induced by use of Babchi seeds as an Ayurvedic remedy for vitiligo. *BMJ Case Rep.* 2014 Aug;2014:bcr2013200958. 10.1136/bcr-2013-200958 Reference Source PMC4127755 25103314

[103] Zhang LL Huang JH : A Case of Drug-Induced Liver Injury Caused by the Combined Medication of Buguzhi Granules and Qubaibabuqi Tablets. *Chin. J. Drug Appl. Monit.* 2018;15(4):246–249.

[104] Li A Gao M Zhao N : Acute liver failure associated with Fructus Psoraleae: a case report and literature review. *BMC Complement. Altern. Med.* 2019 Apr;19(1):84. 10.1186/s12906-019-2493-9 30975110 PMC6458792

[105] Guo Z Li P Wang C : Five Constituents Contributed to the Psoraleae Fructus-Induced Hepatotoxicity via Mitochondrial Dysfunction and Apoptosis. *Front. Pharmacol.* 2021 Dec;12. 10.3389/fphar.2021.682823 34950022 PMC8688997

[106] Li A Ma N Zhao Z : Glycyrrhetinic acid might increase the nephrotoxicity of bakuchiol by inhibiting cytochrome P450 isoenzymes. *PeerJ.* 2016;4(11):e2723. 10.7717/peerj.2723 27904813 PMC5126668

