# Supplementary material for: Bakuchiol, a natural constituent and its pharmacological benefits
Source: F1000Res. 2023 Nov 7;12:29. Originally published 2023 Jan 9. [Version 2] doi: 10.12688/f1000research.129072.2 (PMC10683784; doi:10.12688/f1000research.129072.2)
Supplement: Peer review history

# Reviewer report: Reviewer response for version 2

**DOI:** 10.5256/f1000research.158328.r221455

**Published:** 2023-11-27

**Authors:** Amit Baran Sharangi

**Affiliations:** 1 Department of Plantation Spices Medicinal and Aromatic Crops, Bidhan Chandra Krishi Viswa Vidyalaya, Nadia, West Bengal, India

I found the authors' compliance to be good. It may now be processed further towards indexing in the journal.

Is the review written in accessible language?

Yes

Are all factual statements correct and adequately supported by citations?

Yes

Are the conclusions drawn appropriate in the context of the current research literature?

No

Is the topic of the review discussed comprehensively in the context of the current literature?

Partly

Reviewer Expertise:

NA

I confirm that I have read this submission and believe that I have an appropriate level of expertise to confirm that it is of an acceptable scientific standard.

# Reviewer report: Reviewer response for version 1

**DOI:** 10.5256/f1000research.141728.r177831

**Published:** 2023-08-31

**Authors:** Naoufal El Hachlafi

**Affiliations:** 1 Universite Sidi Mohamed Ben Abdallah, Fes, Fes-Boulemane, Morocco

The manuscript entitled: "Bakuchiol and its pharmacological benefits", presents interesting and valuable work, which is within the scope of the journal ****F1000Research. It is well-written. However, some minor corrections are needed to make this paper accepted for indexing. I encourage the authors to consider making the necessary changes.

**General Considerations**

 The authors should carefully proof-read the entire manuscript to minimize typographical errors, especially with spellings, punctuation, unnecessary capitalizations, spaces, and units, as well as to ensure uniform expression of various special characters and abbreviations, terms, and phrases.

**Results and discussion**

 The description of the results is clear; however, it would be helpful to provide more details in tables taken into account different *in vitro *and *in vivo *clinical studies carried out in this subject.

**Conclusion**

 Conclusion should be improved by adding concluding remarks and future direction for further investigation in the field.

**References**

 Ensure all references are relevant and up-to-date. It's essential to cite the most recent and pertinent research in the field to demonstrate a sound understanding of the current state of knowledge.

Is the review written in accessible language?

Yes

Are all factual statements correct and adequately supported by citations?

Yes

Are the conclusions drawn appropriate in the context of the current research literature?

Yes

Is the topic of the review discussed comprehensively in the context of the current literature?

Yes

Reviewer Expertise:

Pharmacology, essential oils, bioactive compounds, antimicrobial, antioxidant

I confirm that I have read this submission and believe that I have an appropriate level of expertise to confirm that it is of an acceptable scientific standard.

# Reviewer report: Reviewer response for version 1

**DOI:** 10.5256/f1000research.141728.r174263

**Published:** 2023-06-30

**Authors:** Amit Baran Sharangi

**Affiliations:** 1 Department of Plantation Spices Medicinal and Aromatic Crops, Bidhan Chandra Krishi Viswa Vidyalaya, Nadia, West Bengal, India

The article entitled "Bakuchiol and its pharmacological benefits" by Nizam et al., is a routine exercise wherein a systematic review approach summarized, analyzed, interpreted and concluded about the therapeutical values of the compound "bachuchiol" from the leaves and seeds of *Psoralea corylifolia* medicinal plants. While the potential of the same is pharmacologically great, the following are some of my observations on the article which needs immediate attention by the authors and rigorous revision before indexing:

1. To apprise the readers about the origin of the compound "Bakuchiol".....ie., whether it is synthetic or natural (plant based).....some appropriate words may be given in the title itself:
  -it may be like Bakuchiol from Psoralea corylifolia L.
  -it may be like Natural bakuchiol, etc
  The above is for clarity in understanding only.
2. In the "Introduction", placement of Ref 17 is probably at a wrong position. 
3. In the "Results", in the first subhead, the word 'material' has been repeated. Here, the last sentence may be separated into two.
4. Therapeutic applications of bakuchiol: This portion seriously lacks in one or two summarized table containing some global research-based interesting information on In vivo Test, In vitro Test, Plant parts used, Visible / projected Effects, Model used(if any), References, etc
5. Just before "Side effects", was it necessary to hyperlink the highlighted words?
6. After "Side effects", some discussion was necessary on the Phytochemical screening and toxicity study of this very medicinal plants or the compound bakuchiol derived from it .....in a different subtitle along with contraindication, if any and of course, supplemented with a well organised table with convincing references was necessary [LD50 (mg/kg), Phytochemical constituents, References in the last column of the table].
7. Instead of Conclusion, it may be replaced with Conclusion and Future research. In that case, there should be some restructuring of the text with inclusion of some promising upcoming areas of futuristic research in this direction.

Is the review written in accessible language?

Yes

Are all factual statements correct and adequately supported by citations?

Yes

Are the conclusions drawn appropriate in the context of the current research literature?

No

Is the topic of the review discussed comprehensively in the context of the current literature?

Partly

Reviewer Expertise:

Herbs, spices, medicinal and aromatic crops

I confirm that I have read this submission and believe that I have an appropriate level of expertise to confirm that it is of an acceptable scientific standard, however I have significant reservations, as outlined above.

# Response

**Published:** 2023-10-31

**Authors:** Md. Kamrul Hasan

**Affiliations:** North South University, Bangladesh

The article entitled "Bakuchiol and its pharmacological benefits" by Nizam et al., is a routine exercise wherein a systematic review approach summarized, analyzed, interpreted and concluded about the therapeutical values of the compound "bachuchiol" from the leaves and seeds of Psoralea corylifolia medicinal plants. While the potential of the same is pharmacologically great, the following are some of my observations on the article which needs immediate attention by the authors and rigorous revision before indexing:

 To apprise the readers about the origin of the compound "Bakuchiol".....ie., whether it is synthetic or natural (plant based).....some appropriate words may be given in the title itself:

 -it may be like Bakuchiol from Psoralea corylifolia L.

 -it may be like Natural bakuchiol, etc.

**Authors’ response**: Thank you. The revised version addressed this issue and fixed.

 The above is for clarity in understanding only.

 In the "Introduction", placement of Ref 17 is probably at a wrong position.

**Authors’ response**: Addressed and fixed. Thank you.

 In the "Results", in the first subhead, the word 'material' has been repeated. Here, the last sentence may be separated into two.

**Authors’ response**: Thank you. Addressed and fixed.

 Therapeutic applications of bakuchiol: This portion seriously lacks in one or two summarized table containing some global research-based interesting information on In vivo Test, In vitro Test, Plant parts used, Visible / projected Effects, Model used(if any), References, etc.

**Authors’ response**: Thank you so much for the suggestion. The authors revised the manuscript as suggested.

 Just before "Side effects", was it necessary to hyperlink the highlighted words?

**Authors’ response**: Thank you. Hyperlink removed.

 After "Side effects", some discussion was necessary on the Phytochemical screening and toxicity study of this very medicinal plants or the compound bakuchiol derived from it .....in a different subtitle along with contraindication, if any and of course, supplemented with a well organised table with convincing references was necessary [LD50 (mg/kg), Phytochemical constituents, References in the last column of the table].

**Authors’ response**: Thank you so much. The authors revised the manuscript by adding a table as suggested.

 Instead of Conclusion, it may be replaced with Conclusion and Future research. In that case, there should be some restructuring of the text with inclusion of some promising upcoming areas of futuristic research in this direction.

**Authors’ response**: Thank you. Addressed and revised.
